# Methodological considerations in cost of illness studies on Alzheimer disease

**DOI:** 10.1186/2191-1991-2-18

**Published:** 2012-09-11

**Authors:** Nagede Costa, Helene Derumeaux, Thomas Rapp, Valérie Garnault, Laura Ferlicoq, Sophie Gillette, Sandrine Andrieu, Bruno Vellas, Michel Lamure, Alain Grand, Laurent Molinier

**Affiliations:** 1 Department of Medical Information, University Hospital of Toulouse, Toulouse, F-31059, France; 2 UMR 1027, INSERM, Toulouse, F-31059, France; 3 UMR 1027, University of Toulouse III, Toulouse, F-31059, France; 4 Universty of Paris Descartes, Paris, 75016, France; 5 Department of geriatric medicine, University hospital of Toulouse, Toulouse, F-31073, France; 6 Department of Epidemiology and Public Health, University Hospital of Toulouse, Toulouse, F-31059, France; 7 EDISS, University of Lyon I, Villeurbanne, F-69100, France

**Keywords:** Alzheimer disease, Cost study, Dementia, Economic, Evaluation, Review

## Abstract

Cost-of-illness studies (COI) can identify and measure all the costs of a particular disease, including the direct, indirect and intangible dimensions. They are intended to provide estimates about the economic impact of costly disease. Alzheimer disease (AD) is a relevant example to review cost of illness studies because of its costliness.The aim of this study was to review relevant published cost studies of AD to analyze the method used and to identify which dimension had to be improved from a methodological perspective. First, we described the key points of cost study methodology. Secondly, cost studies relating to AD were systematically reviewed, focussing on an analysis of the different methods used. The methodological choices of the studies were analysed using an analytical grid which contains the main methodological items of COI studies. Seventeen articles were retained. Depending on the studies, annual total costs per patient vary from $2,935 to $52, 954. The methods, data sources, and estimated cost categories in each study varied widely. The review showed that cost studies adopted different approaches to estimate costs of AD, reflecting a lack of consensus on the methodology of cost studies. To increase its credibility, closer agreement among researchers on the methodological principles of cost studies would be desirable.

## Review

### Introduction

Cost-of-Illness (COI) studies aim to identify and measure all the costs of a disease [1]. They describe and estimate the economic burden of a specific disease to a society, and therefore the savings that could be done if the disease were to be eradicated [2]. COI studies as decision making tool has been debated, but they may be useful by educating, informing and enlightening policy makers in planning and financing [3]. COI studies are particularly relevant in chronic diseases that weigh heavily on health expenditures. Dementia is characterized by a gradual and irreversible impairment of the intellect, memory, and personality. Alzheimer disease (AD) accounts for 60% to 80% of all dementia cases and its prevalence will increase with the life expectancy growth [4]. There are 35, 6 million demented people in 2011, increasing to 115,4 million by 2050 [5]. Disease worldwide costs were US$ 604 billion in 2011, which 84% were attributable to informal and formal costs. COI studies are the initial step in an economic evaluation. Few cost-effectiveness analyses in AD drug treatment show divergent results in costs saving [6,7], probably because of different methodologies. So, it is necessary to accurately assess AD costs to show the potential economic impact of new therapeutic or preventive strategies. Three articles were previously published on the methodology of AD costs [8-10], but they were either focus on European studies including other dementia (not on AD specifically), or focus on informal costs and not focus on COI studies. This study aims to review relevant published AD COI studies, to analyze the methods used and to identify the points that should be improved in order to obtain convincing results from a methodological perspective. First, we provided a general description of the COI method. And then, we systematically reviewed AD costs studies, analyzing the different methods used.

### Methods

#### Cost study

To conduct a COI study, it is necessary to define disease, epidemiological approach, type of costs, and study perspective. Subsequently, resource consumption data and unit costs can be gathered, and the results presented and methodically discussed, in conjunction with sensitivity analysis to test their robustness. A checklist (Table 1), containing its items, was developped on the model described by Drummond et al. [11] and adapted to COI by Molinier et al. [12]. An equal weight was given to each item. The final score was the sum of the eleven individual items.

**Table 1:** Answers to the methodological questions by study

	**All studies**			**Lopez Bastida et al. **[13]			**Coduras et al. **[14]			**Rigaud et al. **[15]			**Kronborg Andersen et al. **[16]			**Cavallo et al. **[17]			**Mesterton et al. **[18]			**Kiencke et al. **[19]			**Leon et al. **[20]			**Hay et al. **[21]			**Rice et al. **[22]			**Leon et al. **[23]			**Ostbye et al. **[24]			**Zencir et al. **[25]			**Wang et al. **[26]			**Suh et al. **[27]			**Beeri et al. **[28]			**Allegri et al. **[29]		
	**Yes**	**P**	**No**	**Yes**	**P**	**No**	**Yes**	**P**	**No**	**Yes**	**P**	**No**	**Yes**	**P**	**No**	**Yes**	**P**	**No**	**Yes**	**P**	**No**	**Yes**	**P**	**No**	**Yes**	**P**	**No**	**Yes**	**P**	**No**	**Yes**	**P**	**No**	**Yes**	**P**	**No**	**Yes**	**P**	**No**	**Yes**	**P**	**No**	**Yes**	**P**	**No**	**Yes**	**P**	**No**	**Yes**	**P**	**No**	**Yes**	**P**	**No**
1 Was a clear definition of the illness given?	10	5	2		P		Yes			Yes			Yes					No		P			P		Yes					No		P		Yes			Yes			Yes			Yes				P		Yes			Yes		
2 Were epidemiological sources carefully described?	13	3	1	Yes			Yes			Yes			Yes			Yes				P		Yes			Yes			Yes			Yes				P		Yes				P		Yes			Yes					No	Yes		
3 Were costs sufficiently disaggregated ?	12	0	5	Yes			Yes					No	Yes			Yes			Yes			Yes					No	Yes			Yes					No			No	Yes			Yes					No	Yes			Yes		
4 Were activity data sources carefully described?	15	1	1	Yes			Yes			Yes			Yes			Yes			Yes				P		Yes			Yes			Yes			Yes					No	Yes			Yes			Yes			Yes			Yes		
5 Were activity data appropriately assessed?	6	9	2	Yes				P			P			P			P		Yes					No	Yes			Yes			Yes				P				No		P			P		Yes				P			P	
6 Were the sources of all cost values analytically described?	10	4	3	Yes			Yes			Yes			Yes				P		Yes					No	Yes			Yes				P			P				No		P		Yes			Yes					No	Yes		
7 Were unit costs appropriately valued?	7	5	5	Yes			Yes				P			P			P		Yes					No	Yes			Yes				P				No			No			No	Yes			Yes					No		P	
8 Were the methods adopted carefully explained?	11	6	0	Yes			Yes			Yes			Yes				P		Yes				P		Yes			Yes				P			P			P		Yes			Yes			Yes				P		Yes		
9 Were costs discounted ?	0	2	15			No			No			No			No			No			No		P				No		P				No			No			No			No			No			No			No			No
10 Were the major assumptions tested in a sensitivity analysis?	3	0	14			No	Yes					No			No			No			No	Yes					No			No			No			No			No			No			No	Yes					No			No
11 Was the presentation of study results consistent with the methodology of study?	13	4	0	Yes			Yes			Yes			Yes			Yes			Yes			Yes				P			P		Yes			Yes				P		Yes			Yes			Yes				P		Yes		
12 Total score by study	100	39	48	8	1	2	9	1	1	6	2	3	7	2	2	4	4	3	7	2	2	4	4	3	7	1	3	7	2	2	5	4	2	3	4	4	2	2	7	5	3	3	8	1	2	8	1	2	3	3	5	7	2	2

Total score by study was the sum of answers; P, Partially.

#### Defining the disease and population

Illness costs widely depend on how the disease is defined. AD diagnosis is based on Alzheimer’s Association criteria (NINCDS-ADRDA) [30] and Diagnostic and Statistical Manual of Mental Disorders criteria (DSM IV) [31]. As costs increase with disease severity [32-35], disease stage must be specified and measured using validated tools, as Clinical Dementia Rate [36] (CDR) or Mini Mental State Examination (MMSE) [37]. Cost components may vary depending on the living conditions (e.g. home, institution), and therefore must be specified [33,38].

#### Epidemiological approach

Prevalence-based COI studies estimate the economic burden to society during a period of time as a result of the prevalence of the disease (e.g. in a given year). Incidence-based studies estimate lifetime costs, and measure the costs of an illness from diagnosis until recovery or another endpoint (e.g. death).

#### Perspective of the analysis and costs assessed

A COI study may be conducted from several perspectives that must be specified to check that relevant costs are included. From the healthcare payer perspective, only direct costs incurred by a payer (e.g. national health insurance) are considered. Indirect costs and the patient “out-of-pocket” must also be included in a study which uses societal perspective.

#### Estimating resource consumption

Resource consumption estimates vary depending on the data available, but validated tools exist to collect them [39]. In prospective COI studies, events have not occurred yet, so data collection is done by the patients’ follow-up, medical records, data from clinical trials and patients or caregiver questionnaires. Conversely, in retrospective COI study, events have already occurred, so data collection must refer to recorded data, either using “Top-down” method (aggregate figures from hospital admissions, mortality, etc.) or “bottom-up” method (by referring to the patients sample record).

#### Valuation of unit costs

The COI is estimated by identifying the cost-generating components and by attributing them a monetary value. This is the opportunity cost, the value of the forgone opportunity to use in a different way those resources that are lost due to illness [11]. Direct and indirect costs should be valued to assess the total economic COI. Direct costs measure the resources used to treat an illness and can be estimated by per capita expenditures, national tariffs, market prices, and published studies. Patient charges and tariffs do not give an accurate estimate of the underlying costs. Market prices can be used to value some cost categories like drugs, rehabilitation items (e.g. eyeglasses, etc.). Direct costs can also be valued with care facilities estimates, through the analytical account system which specified distribution properties. Indirect costs measure the loss of productivity, the effect of the illness on the patient or caregiver abilities to work. Three methods are used to value indirect costs: the Human Capital Approach (HCA) [40], the Friction Cost Method [41] and the Willingness to pay approach [42]. Informal care is an unpaid care often provided by relatives and plays a substantial role in the AD patient’s total care. Two methods are mainly used to value the shadow price of informal care time. The opportunity cost approach values the opportunity forgone as a result of caregiving and the replacement cost approach values the caregiving time spent at a price of a close substitute [9,43,44].

#### Discounting costs

Discounting captures individual preferences for income today rather than income in the future and is frequently applied when COI studies are considered over several years. In the USA, they estimate the discount rate at 3% [45]. The following equation is applied to estimate costs:

(1) $$C_{a}=C_{t}{\overset{t}{\underset{n=1}{\sum }}\left(1+r\right)}^{-n}$$

Where: *C*_*a*_ = present value of cost strategy, *C*_*t*_ = value of cost strategy in year t, r = discount rate, t = time period.

#### Sensitivity analysis

Sensitivity analysis is recommended because COI studies contain uncertainties. It allows testing the robustness of the results by varying in range key variables (e.g., prevalence, unit costs, etc.) [46]. It seems more credible to present a range of possible illness costs.

#### Presentation of results

The presentation of COI results should be consistent with collected data and should disaggregate results into as many components as possible with full explanations given for clarity (Table 1).

#### Literature review

##### Study selection

**Figure 1:**
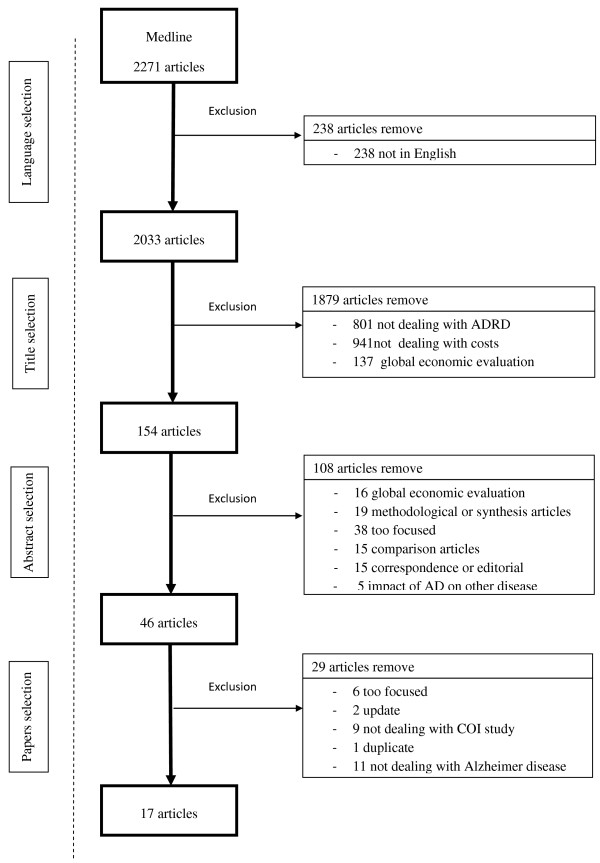
Literature search and selection process.

A bibliographic search was performed on an international medical literature database (Medline). All studies which assessed the economic burden of AD were selected. To be exhaustive, eight keywords combinations were employed: “Alzheimer disease” AND “Cost of illness”; “Alzheimer disease” AND “Economic evaluation”; “Alzheimer disease” AND “Cost study”; “Alzheimer disease” AND “Cost analysis”; “Dementia” AND “Cost of illness”; “Dementia” AND “Economic evaluation”; “Dementia” AND “Cost study”; “Dementia” AND “Cost analysis”. This search provided us 2271 papers. We kept the 2033 papers written in English. Among them, we selected articles whose title contained “Dementia” *or* “Alzheimer disease” (801 papers were removed) AND “Costs” *or* “Expenses” *or* “Economic” *or* “Burden” (another 941 papers were removed). This study focused on the methodology used to estimate AD costs, 137 papers were removed because they were identified as global economic analyses. A hundred and fifty four abstracts were first selected and 46 of them underwent a subsequent full paper reading, thus providing 17 articles. Figure 1 illustrates the literature search, selection process, and presents reasons for excluded studies.

##### Study review

A systematic review was performed. One author (N. Costa) selected abstracts. Six methodologists read the 46 papers retrieved by the search strategy and reviewed the 17 selected papers. With the key methodological points identified in the first part of the paper, they asked questions based on existing checklists for full economic evaluations [47]. The objective was not to establish a criteria hierarchy by using different weights, but to use these criteria to analyze the methods used. Each study was assessed separately by the reviewers. Finally, a consensus was reached by discussion. Then, all authors, both clinicians and methodologists, discussed the results.

### Results

Seventeen studies met our criteria (Tables 2, 3). Seven studies were conducted in Europe [13-19], 5 in North America [20-24], 4 in Asia [25-28] and 1 in South America [29]. Fifteen studies selected a sample ranging in size from 42 to 21512 patients [13-20,22-29].

**Table 2:** Cost of illness studie's characteristics in Alzheimer disease

**Study**	**Country**	**Type of helthcare system (insurance)**	**Year of valuation**	**Currency**	**Perspective**	**Design of cost analysis**	**Sample size**	**Type of setting**	**Follow-up (months)**	***Total annual cost per patient (US$)***
Lopez Bastida et al. [13]	Spain	Public social	2001	€	Societal	Prosepective	237	At home	12	***37,881***
Coduras et al. [14]	Spain	Public social	2006	€	Societal	Prospective multicentre	560	At home and in institution	12	***22,558***
Rigaud et al. [15]	France	Public social	1996	€	Societal	Retrospective single centre	48	At home	6	***31,153***
Kronborg Andersen et al. [16]	Denmark	Public social	1997	DKK	Societal	Prospective single centre	164	At home and in institution	12	***17,078***
Cavallo et al. [17]	Italy	Public and private	1995	₤	Family	Prospective single centre	423	At home	NS	***52,954***
Mesterton et al. [18]	Sweden	Public social	2007	SEK	Societal	Prospective multicentre	233	At home and in institution	1	***46,956***
Kiencke et al. [19]	Germany	Public health	2005	€	Healthcare payer	Decision model	21512	NS	60	***11,786***
Leon et al. [20]	USA	Private	1996	US$	Societal	Prospective multicentre	150	At home and in institution	1	***18,804***
Hay et al. [21]	USA	Private	1983	US$	Societal	Retrospective	NS	At home and in institution	Lifetime	18,517*
Rice et al. [22]	USA	Private	1990	US$	Societal	Prospective multicentre	187	At home and in institution	12	51,905*
Leon et al. [23]	USA	Private	1996	US$	Societal	Prospective multicentre	679	At home and in institution	NS	***27,672***
Ostbye et al. [24]	Canada	Public social	1991	CAN$	Societal	Not specify	10263	At home and in institution	NS	13,900*
Zencir et al. [25]	Turkey	Public and private	2003	TRY	NS	Prosepective	42	At home	3	***3,492***
Wang et al. [26]	China	Public social insurance	2006	RMB	NS	Prospective single centre	66	NS	12	2,935*
Suh et al. [27]	Korea	Private	2002	₩	Societal	Decision model	NS	At home and in institution	12	***11,389***
Beeri et al. [28]	Israel	Public social	1999	NIS	Societal	Prospective multicentre	121	At home and in institution	6	19,893*
Allegri et al. [29]	Argentina	Public and private	2001	$Ar	Societal	Retrospective	100	At home and in institution	3	***7,709***

NS: not specify; NA: not available, * Net Costs.

All costs are in US$ (1€ = 1,36491 US$, 1 CAN$ = 0,970458 US$, 1 DKK = 0,183360 US$, 1 RMB = 0,156987 US$; October 11, 2011) SEK, Swedish Crown; RMB, Yuan Renminbi; ₩, Won; $Ar, Argentine Peso; NIS, New Israeli Shekel; TRI, Turkish Lira; CAN$, Canadian Dollar; DKK, Danish Crown; €, Euro).

**Table 3:** Total annual costs disaggregation

**Study**	***Total annual cost per patient (US$)***	**Direct medical costs**	**Inpatient**	**Outpatient**	**Medication**	**Specialized institution**	**Other**	**Direct non medical costs**	**Home help**	**Nursing home**	**Transportation**	**Other**	**Informal costs**	**Indirect costs**
Lopez Bastida et al. [13]	***37,881***	**4,848**	924	844	2,468	301	311	**2,306**	2,223	NA	83	NA	**29,884**	**843**
Coduras et al. [14]	***22,558***	**4,744**	144	503	2,137	1,97	NA	**5,798**	4,66	1,138	NA	NA	**12,016**	**NA**
Rigaud et al. [15]	***31,153***	**6,663**	NS	NS	NS	NS	NA	**5,632**	5,632	NS	NS	NA	**18,858**	**NA**
Kronborg Andersen et al. [16]	***17,078***	**4,357**	4,11	247	NA	NA	NA	**12,721**	12,663	NA	NA	58	**NA**	**NA**
Cavallo et al. [17]	***52,954***	**2,722**	NA	NA	NA	2,722	NA	**5,496**	5,496	NA	NA	NA	**44,736**	**NA**
Mesterton et al. [18]	***46,956***	**3,155**	1,067	1,118	970	NA	NA	**39,373**	6,487	32,886	NA	NA	**4,428**	**NA**
Kiencke et al. [19]	***11,786***	**11,786**	2,889	1,449	2,126	NS	677	**NA**	NA	NA	NA	NA	**NA**	**NA**
Leon et al. [20]	***18,804***	**7,284**	NS	NS	NS	NA	NA	**NA**	NA	NA	NA	NA	**11,52**	**NA**
Hay et al. [21]	18,517*	**2,292**	756	1,292	244	NA	NA	**7,285**	1,774	5,326	167	18	**9**	**NA**
Rice et al. [22]	51,905*	**22,176**	1,072	545	301	19,521	737	**9,699**	9,585	NA	NA	114	**20,03**	**NA**
Leon et al. [23]	***27,672***	**21,924**	NS	NS	NS	NS	NS	**NA**	NA	NA	NA	NA	**5,748**	**NA**
Ostbye et al. [24]	13,900*	**NS**	NS	NS	NS	NS	NS	**NS**	NS	NS	NA	NS	**NS**	**NA**
Zencir et al. [25]	***3,492***	**2,128**	NA	37	2091	NA	NA	**NA**	NA	NA	NA	NA	**1,364**	**NA**
Wang et al. [26]	2,935*	**863**	29	32	802	NA	11	**431**	373	NA	NA	27	**1,63**	**NA**
Suh et al. [27]	***11,389***	**4,394**	NS	NS	NS	NA	NA	**445**	445	NA	NA	NA	**6,55**	**NA**
Beeri et al. [28]	19,893*	**3,974**	1,749	1,63	584	NA	11	**9,326**	1,822	7,504	NA	NA	**6,593**	**NA**
Allegri et al. [29]	***7,709***	**3,389**	525	280	2,389	NA	195	**2,488**	187	2,301	NA	NA	**1,832**	**NA**

NS: not specify; NA: not available, * Net Costs.

All costs are in US$ (1€ = 1,36491 US$, 1 CAN$ = 0,970458 US$, 1 DKK = 0,183360 US$, 1 RMB = 0,156987 US$; October 11, 2011).

SEK, Swedish Crown; RMB, Yuan Renminbi; ₩, Won; $Ar, Argentine Peso; NIS, New Israeli Shekel; TRI, Turkish Lira; CAN$, Canadian Dollar; DKK, Danish Crown; €, Euro).

### Defining the disease and population

AD was defined with NINCDS-ADRDA criteria for 4 studies [20,23,28,29], with DSM criteria for 2 studies [25,26], with both criteria for 4 studies [14-16,24] and with ICD-10 for another study [19]. Six studies did not specify the criteria used [13,17,18,21,22,27]. Disease severity was measured with MMSE in 9 studies [14,15,18,22,24-28], with CDR in 3 studies [13,16,23], and with both tests for 2 studies [20,29]. Disease severity was not specified in three studies [17,21,23]. Four studies included community dwelling patients [13,15,17,25] and 11 studies included both patients in community and in institution [14,16,18,20-24,27-29]. The place of living was not specified in 2 studies [19,26].

Thirteen studies specified the mean age of the sample of patients ranging from 70,5 to 81,8 years [13-20,22,25,26,28,29].Two studies included patients aged from 50 to over 80 years [21,24].

#### Perspective of the analysis and costs assessed

The adopted perspective was the society in 13 studies [13-16,18,20-24,27-29] and respectively the family and the healthcare payer in two studies [17,19]. Fourteen studies quantified direct medical and non-medical costs and informal costs [14,15,17,18,20-29]. Two studies did not include inpatient costs [17,25]. Informal costs were defined as direct non-medical costs in seven studies [14,15,17,19,22,23,25] and as indirect costs in seven studies [18,21,24,26-29]. One study quantified all the costs (i.e. direct, informal and indirect) [13], indirect costs were defined as patient early retirement and informal costs as direct non-medical costs. Danish study quantified only direct medical and non-medical costs [16]. German study quantified only direct medical costs [19].

#### Estimating resource consumption

Three studies estimated resource consumption retrospectively [15,21,29]. Two of them used bottom-up approach to gather activity data through questionnaires [15,29]. The other used a top-down approach, using published national indicators, national surveys and published studies [21]. Eleven studies estimated resource consumption prospectively [13,14,16-18,20,22-24,26,28]. Two studies used mainly the Resource Utilisation in Dementia (RUD) [48] to gather activity data [14,18], completed with report forms, medical records and questionnaires on 560 patients in the Spanish study [14]. Nine studies gathered activity data mainly with questionnaires [13,16,17,20,22,23,25,26,28]. In the Danish study, 164 AD couple (i.e. patients/caregivers) was interviewed at home about Activities of Daily Living (ADL), use of health care and community services [16]. Two US studies measured direct costs using caregiver’s interviews and Medicare Current Beneficiary Survey (MCBS) [13,23]. Rice et al. gathered activity data through monthly caregiver’s telephone interviews, billing records and with calendar given to caregiver at the baseline visit [23]. Israeli study used a baseline questionnaire to record time spent on caring and use of goods and services, and secondly recorded the same items with 5 monthly telephone interviews [28]. Two studies recorded data via mailed questionnaires filled out by caregivers [13,17]. Turkish study used a questionnaire and daily time sheets for caregiving time [25]. Wang et al. interviewed 66 AD couples for filling out the resource use’s questionnaire [26]. Two studies used decision modelling [19,27] and estimated resources with published sources and national surveys [27] or with data extractions of a German retrospective analysis [19]. One study did not precise the approach used to gather activity data [24]. Eight studies specified the number of caregiver included, several for 4 studies [13,15,17,22] and only one for others [18,23,25,28]. Eight studies recorded AD net costs [15,16,21,22,24,26,28,29] either by subtracting healthy patient costs or by asking AD couple about resource’s used exclusively for AD.

The follow-up period was the lifetime in the study which adopted the incidence-based approach [21] and was one year in six studies [13,14,16,22,26,27], but was frequently reduced to six, three or one month [14,18,20,25,28,29]. One study used a sixty months follow-up period [19].

#### Valuation of unit costs

Direct costs were estimated from published data, national estimates and Medicare-over charges in one American study [21], and from unit costs using the MCBS and national estimates for adult day care in another one [20]. Rice et al. used charges and bills provided by the caregivers [22]. Two Spanish studies used the Spanish database on medical costs (SOIKOS), patient’s reports and the Spanish Vademecum to estimate unit costs [13,14]. Direct unit costs were based on reimbursement tariffs used by the French social health insurance and on the French Disease related group (DRG) for inpatient care [15]. The Danish study used the reimbursement tariffs of social insurance, hospital accounts, gross wage rates of professionals and amortization procedure to value direct costs [16]. Institutional costs were valued with the average cost per day without food and beverage. Zencir et al. valued unit costs with the average price of public and private physician visits and with the average price per day for medication [25]. In the Chinese study, unit costs were valued with drugs prices, transportation reimbursements and local fees for home help [26]. Tariffs of social health insurance and full drugstore prices were used to unit costs valuation in the Argentinian study [28]. Meserton et al. valued unit costs with ward-specific per diem costs from regional price, the lowest available price for medication and with per visit costs [21]. For residential care, the number of days in institution was multiplied by the corresponding unit costs. Two studies used national estimates to value unit costs [23,27]. No information relating to direct costs valuation was reported in four studies [17-19,24]. Twelve studies used the replacement cost approach to value informal care [13,15,17,20-26,28,29]. Seven studies used national estimates of a close substitute [17,20-23,25,29]. Among these, 5 studies used different national wages to value different caregiver’s activities [17,20,22,23,28]. Israeli study obtained different hourly wage rates for each activity from the Central Bureau of Statistics [28] and Leon et al. used hourly wage of home health aids to value ADL time and homemaker’s hourly wage to value IADL time [23]. Two studies used hospital nurse hourly wage [21,25], another one, local wage rate without specifying the type of professional caregiver [26] and the Canadian study used published data [24]. Three studies did not specify the sources used to value informal care time [13,15,29]. Nevertheless, one study used professional caregiver monthly salary [29], another the cost per hour of a domestic cleaner gross wage [13] and the last one the average between housekeeping and paid assistance wage [15]. One study chose opportunity cost approach to value informal care, using the mean informal caregiver own salary [14]. Both approaches were used in one study [27]. The time spent by working caregivers was valued with national estimates and the time spent by working caregivers was valued with the own caregivers salaries. Opportunity cost and revealed preference approaches were used to value informal costs in one study [18]. Caregiving time was valued either with the HCA (working caregivers) or with a monetary estimate value of one hour of leisure time (not working caregivers). One study valued indirect costs with national estimates on employment and wages [13].

#### Discounting costs

One study has discounted costs without specifying the discounting rate [21]. German study performed costs discounting in the sensitivity analysis [19]. All the other studies used a short follow-up period and had no need to discount costs.

#### Sensitivity analysis

Only three studies performed a sensitivity analysis [14,27,29]. One study analyzed the impact of informal costs variation [14], another one the variation of AD sufferers’ proportion according to the need of full time care level [27], and the last one the discounting of the incurred costs [19].

#### Presentation of results

Most studies presented their results clearly. They were mainly well explained and consistently set out in relation to the methods adopted. Five studies did not sufficiently disaggregate costs, so the information strength was reduced (Table 3) [15,20,23,24,27]. All studies presented results in terms of cost per patient. Four studies proposed also AD total costs for the country [13,23,24,27]. According to the key methodological points, we have drafted a checklist of questions related to the eight items analyzed (Table 1). For 9 studies, the answers of at least seven to eleven questions were “yes” [13,14,16,18,20,21,26,27,29].

## Conclusion

This study reviewed seventeen COI studies on AD with the main goal of analyzing the various methodologies. According to the key methodological points, nine studies scored “yes” on the majority of the questions [13,14,16,18,19,21,26,27,29].

In this review, annual cost per patient varies from $2,935 to $52,954, confirming the costly character of AD. Nevertheless, commenting on these quantitative results is a problem because different approaches have been adopted. Informal care time varies widely according to the tool used. Validated instruments such as RUD, Caregiver Activity Survey (CAS) or Caregiver Activity Time Survey (CATS) exist to estimate informal care time [48-50]. Most often, time spent in ADL and IADL was used but they measure dependence and not specifically AD caregiving time. Opportunity cost underestimates the time of women, elderly and minority that suffer from discrimination in labour market [51], and does not allow the valuation of different informal caregiving activities. These activities change can be considered with the replacement cost approach. Informal costs vary with the number of caregiver included. Informal costs can be 8 times higher if several caregivers are included rather than just one. AD informal costs have to be rigorously quantified because they represent 36% to 85% of total costs [9].

Unlike clinical trial results, it is very difficult to generalize results of economic studies conducted in different countries. Economic results are difficult to compare because of monetary issues (i.e. fluctuating exchange rates, purchasing powers of currencies). According to the World Bank classification [52], 3 studies in this review were conducted in upper middle income economies [25,26,29] and presented a mean annual cost for an AD patients which is 5 times lower than in studies conducted in high income economies. Purchasing Power Parity (PPP) use could help results comparison because it eliminates price levels differences between countries [53].

Domestic characteristics also affect resource consumption and unit costs, including differences in clinical practice and healthcare system framework. For example, medication costs can vary between studies because of the use of tariffs in solidarity systems which are not comparable to free prices in private systems.

Follow-up periods found in this review were often less than one year, which is a short period to assess chronic disease costs. However, data collection over a long period is difficult so the use of models could compensate this difficulty.

Some limitations are present in this review as only English papers were selected, which restricted our sample. Another limitation is based on the lack of items weighting (Table 1). It is likely that results are more significantly affected by some items than others. Further works must be performed in this area.

Nevertheless, this study built an inventory on the methodology used to analyze AD costs and helped in better understanding the reasons of disparate results between studies.

COI results are the basis for economic evaluations and provide information for models that is a part of any efficiency evaluation [45]. Nevertheless, an insufficient description of methods may lead to misunderstandings. COI studies identified in this review highlight the poor consensus of methodological approaches. Medical journal should encourage researchers to give clear descriptions and discuss limitations, and a further effort should be made to validate methodology. The definition of standards, with a large consensus in the methodology selected to conduct this studies, should be a major concern for the scientific community. A collective awareness about disease burden exists between economists, policies and caregivers that may lead to relevant decision making. COI studies can serve as a basis for projecting disease expenses, and thus allow adapting medical and social disease management in order to control AD costs.

## Abbreviations

AD: Alzheimer disease; ADL: Activity of Daily Living; CAS: Caregiver Activity Survey; CATS: Caregiver Activity Time survey; COI: Cost of illness study; CDR: Clinical Dementia Rate; DSM: Diagnostic and Statistical Manual of Mental Disorders; DRG: Disease Related Group; HCA: Human Capital Approach; IADL: Instrumental Activity of Daily Living; MCBS: Medicare Current Beneficiary Survey; MMSE: Mini Mental State Examination; N: No; NINCDS-ADRDA: National Institute of Neurological and Communicative Disorders and Stroke and the Alzheimer's Disease and Related Disorders Association; NA: Not Applicable; NS: Not Specified; NM: Not Measured; MNS: Measured but Not Specified; P: Partially; PPP: Purchasing Power Parity; RUD: Resource Utilization in Dementia; Y: Yes.

## Competing interests

The authors declare that they have no competing interests.

## Authors’ contributions

NC supervised the project and its implementation, conducted the literature review, drafted the article and approved his final version. LM designed the study and helped supervise the project and its implementation, conducted the literature review, drafted the article and approved his final version. HD and TR helped to implement the project, conducted the literature review, reviewed the article and approved final version.VG and LF helped conduct the literature review, reviewed the article and approved his final version. SG, SA, ML, AG and BV helped the interpretation of data, reviewed the article and approved his final version. All authors read and approved the final manuscript.

## Financial support

This study was supported by a grant from the French Ministry of Health (PHRCN 2008, 08 111 01).
